# What is Global Health Equity? A Proposed Definition

**DOI:** 10.5334/aogh.3754

**Published:** 2022-07-04

**Authors:** Ella August, Lia Tadesse, Marie S. O’Neill, Joseph N. S. Eisenberg, Rex Wong, Joseph C. Kolars, Abebe Bekele

**Affiliations:** 1 University of Michigan School of Public Health, 1415 Washington Heights, Ann Arbor, Michigan, US; 2 Ministry of Health, Ethiopia. Sudan St, Addis Ababa, ET; 3 University of Global Health Equity, Kigali Heights, RW; 4 University of Michigan Medical School, Department of Learning Health Sciences, 1111 E. Catherine St., Ann Arbor, MI, US

**Keywords:** Global health equity, global health, health equity, racism, partnerships, colonialism, determinants of health

## Abstract

The term “global health equity” has become more visible in recent years, yet we were unable to find a formal definition of the term. Our Viewpoint addresses this gap by offering a discussion of this need and proposing a definition. We define global health equity as mutually beneficial and power-balanced partnerships and processes leading to equitable human and environmental health outcomes (which we refer to as “products”) on a global scale. Equitable partnerships actively work against racism and supremacy. Such partnerships foster processes with these same dynamics; for example, sharing lead authorship responsibilities with meaningful roles for host country researchers to frame relevant questions and to provide context and interpretation for the research findings. Equitable products, such as access to technology and tailored delivery of interventions effective in the specific context, are the fruits of these partnerships and processes.

Terminology describing global health activities has changed and expanded over past decades as our values in this space have changed [1]. Early on, tropical medicine focused on maintaining a labor force in the colonial tropics. As the approach to and nature of global health activities changed, the language changed. International health largely replaced the term tropical medicine with a focus on high-income countries helping low and middle-income countries; this highlighted the power disparity between the two regions [2,3]. Eventually, the term global health came into favor, reflecting new emphases. Koplan et al.’s widely cited definition mentions equity: “global health is an area for study, research, and practice that places a priority on improving health and achieving equity in health for all people worldwide [4].” They specify that global health involves a multidisciplinary approach, stating that it “emphasizes transnational health issues, determinants, and solutions; involves many disciplines within and beyond the health sciences and promotes interdisciplinary collaboration; and is a synthesis of population-based prevention with individual-level clinical care.” More recently, the shift to the term global health includes a greater awareness about the environment and climate change as global health concerns.

The term global health equity has increasingly appeared in the literature, and the organizations, centers, fellowships, and degree programs with “global health equity” in their name are much more visible than even five years ago. While we were unable to find a formal definition of global health equity, there has been discussion of the term. Some have argued that the term is simply a “rebranding” of global health to distance it from its colonial roots and power disparities [5].

Is the term global health equity meaningfully different than global health? Is the addition of “equity” necessary? We argue here that adding the word equity is important, as our language has the power to shape our future discourse and actions [6,7]. We assert that global health equity is inextricably linked to power-balanced and mutually beneficial partnerships and processes. Additionally, we contend that only these types of partnerships and processes can truly lead to equitable health (including health promotion) outcomes, what we refer to here as “products.” To both reflect our current values and support progress in the global health space, we offer a definition of global health equity that incorporates partnerships, process, and products below.

Equity is a central goal of public health, global health, global social medicine, and planetary health, and in fact many definitions of these terms include the word equity. Health equity has been defined in different ways, but generally refers to the absence of unfair and avoidable differences in health among population groups defined socially, economically, demographically, and/or geographically [8]. As often seen in definitions of equity, the focus here is on the product (health outcomes).

Major gaps in equity are pervasive, and we highlight two current examples here. Early efforts at containing COVID, one of the worst pandemics in history, highlighted extreme disparities in vaccine coverage between high- and low-income countries. Vaccine apartheid left low-income countries with less than 1% coverage early in 2021 while high-income countries leveraged their neocolonial negotiating power, global policy might, and financial resources to acquire more than double the doses needed to cover their citizens [9]. Early on, Canada secured nearly nine vaccine doses per person [10]. While equitable vaccine access (the product) has been discussed, two essential aspects—partnerships and processes—have been largely overlooked.

Another example pertains to disparities in access to surgical care. More than five billion people in the world do not have timely access to safe surgery and anesthesia, and only 6% of the 313 million surgical procedures are performed in low- and middle-income countries (where these surgical procedures are needed the most) [11]. In addition, low-income and lower-middle-income countries, representing 48% of the global population, have 20% of the global surgical workforce, or 19% of all surgeons, 15% of anesthesiologists, and 29% of obstetricians. Again, while this outcome disparity has been recognized, focus on partnerships and processes has been lacking.

As Abimbola and Pai discuss, concern for global and international health was originally in service of colonization, and global health efforts and organizations today are characterized by supremacist structures and leadership [12]. Decolonization and the eradication of racism are ultimate goals that will transform the way global health activities are practiced. Engaging in practices and reflection that lead toward global health equity in the current moment will move us closer to these goals [13].

In building our definition, we move beyond the sole focus on products to emphasize partnerships and processes. To that end, we highlight three principles that have formed our definition. First, partnerships are a key launch point for global health equity. Although Koplan et al [4]. did not explicitly refer to partnerships in the above-quoted definition of global health, their paper does touch on the importance of “real partnership[s]” and “a pooling of experience and knowledge” with a “two-way flow between developed and developing countries.” Importantly, we depart from Koplan et al.’s framing that global health partnerships are necessarily between low-income countries and high-income countries. Such partnerships also involve collaborations within low-income countries and within high-income countries without the involvement of foreign actors. Regardless of where the collaborators are from, engaging in partnerships that are equal in power and benefit, and equitable in sharing resources, is critical to the success of global health activities.

We appreciate the challenges of developing equitable partnerships in the context of existing power imbalances [14]. Despite these challenges, we believe it is a step forward to make equitable partnerships a standard from which global health work is funded and implemented. Successful global health partnerships that actively work against racism and supremacy are achieved by offering mutual respect, engaging in activities with mutual benefit, developing trust, practicing good communication, and establishing clear partner roles and expectations [15].

Second, we assert that mutually beneficial and power-balanced partnerships foster processes with these same dynamics. For example, one proxy metric for such a research partnership is the proportion of academic manuscripts led by host country investigators [16]. Researchers who are part of a collaboration guided by these principles have the training and opportunity to lead manuscripts [17]; with this role comes decision making power. Including host country authors enhances a team’s ability to frame relevant questions, providing meaningful context and interpretation for the research findings [18]. The use of policies such as requiring author teams to submit reflexivity statements with their journal submissions describing the ways in which equity has been promoted in their partnership can help make these practices more standard [13,17].

Third, equitable products are the fruits of the partnerships and processes described above. Balanced partnerships and intellectual exchange are the backbone of effective processes leading to equitable products [19,20,21]. For example, partnerships guided by community-based participatory research principles improve the rigor (the practice and promotion of good science), relevance (the quality and appropriateness of the research questions posed) and reach (the degree to which knowledge is disseminated to diverse audiences and translated to useful tools for the scientific, regulatory, policy and lay arenas) of research [21].

Our definition of global health equity recognizes “equitable health” as a key outcome of global health activities, as acknowledged in Koplan et al.’s definition [4]. Accessibility to health and health services is a core aspect of health equity [22]. A health outcome “product” that goes beyond a focus on humans is the health of the environment, which is linked to human health and wellbeing in a multitude of ways. These linkages have been the impetus for movements including One Health [23], environmental justice, and planetary health that encompass aspects of all of these movements, and we include this in our concept of global health equity.

Therefore, we define global health equity as mutually beneficial and power-balanced *partnerships* and *processes* leading to equitable human and environmental health *products* on a global scale (see Figure 1). Although many existing definitions of global health mention one of the three, few mention them all, and their presence in our proposed definition reinforces the idea that global health equity requires all three to be in place. Ultimately, a commitment to all three components of our definition is necessary to move toward health equity for all.

**Figure 1:**
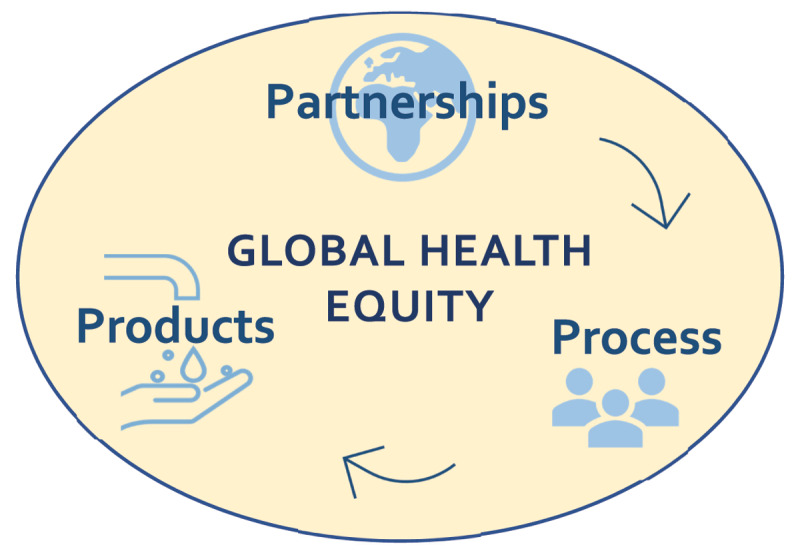
Depiction of the dynamic relationship between partnerships, processes, and products in our definition of global health equity: “Mutually beneficial and power-balanced *partnerships* and *processes* leading to equitable human and environmental health *products* on a global scale.”

## References

[1] Salm M, Ali M, Minihane M, Conrad P. Defining global health: Findings from a systematic review and thematic analysis of the literature. BMJ Glob Heal. 2021; 6(6). DOI: 10.1136/bmjgh-2021-005292 PMC8183196 34083243

[2] Abimbola S. On the meaning of global health and the role of global health journals. Int Health. 2018; 10(2): 63–65. DOI: 10.1093/inthealth/ihy010 29528402

[3] Greene JA, Basilico MT, Kim H, Farmer P. Colonial medicine and its legacies. In: Farmer P, Kleinman A, Kim J, Basilico M, (eds.), Reimagining global health: An introduction. University of California Press; 2013.

[4] Koplan JP, Bond TC, Merson MH, et al. Towards a common definition of global health. Lancet. 2009; 373(9679): 1993–1995. DOI: 10.1016/S0140-6736(09)60332-9 19493564 PMC9905260

[5] Wollner E, Law T, Sullivan K, Lipnick MS. Why every anesthesia trainee should receive global health equity education. Can J Anesth. 2020; 67(8): 924–935. DOI: 10.1007/s12630-020-01715-3 32483743

[6] Hommes F, Monzó HB, Ferrand RA, et al. The words we choose matter: recognising the importance of language in decolonising global health. Lancet Glob Heal. 2021; 9(7): e897–e898. DOI: 10.1016/S2214-109X(21)00197-2 34143986

[7] Richardson ET. Epidemic illusions: On the coloniality of global public health. The MIT Press; 2021. DOI: 10.7551/mitpress/12550.001.0001

[8] Pan American Health Organization. PAHO Heath Equity. https://www3.paho.org/hq/index.php?option=com_content&view=article&id=5586:health-equity-egc&Itemid=0&lang=en.

[9] Harman S, Erfani P, Goronga T, Hickel J, Morse M, Richardson ET. Global vaccine equity demands reparative justice-not charity. BMJ Glob Heal. 2021; 6(6): 4–7. DOI: 10.1136/bmjgh-2021-006504 PMC8215249 34919057

[10] Mullard A. How COVID vaccines are being divvied up around the world. Nature. Published online November 2020. https://www.nature.com/articles/d41586-020-03370-6. DOI: 10.1038/d41586-020-03370-6 33257891

[11] Meara JG, Leather AJM, Hagander L, et al. Global Surgery 2030: Evidence and solutions for achieving health, welfare, and economic development. Lancet. 2015; 386(9993): 569–624. DOI: 10.1016/S0140-6736(15)60160-X 25924834

[12] Abimbola S, Pai M. Will global health survive its decolonisation? Lancet. 2020; 396(10263): 1627–1628. DOI: 10.1016/S0140-6736(20)32417-X 33220735

[13] Kumar M, Atwoli L, Burgess RA, et al. What should equity in global health research look like? Lancet. 2022; 6736(22): 18–20. DOI: 10.1016/S0140-6736(22)00888-1 35597247

[14] Farmer P, Kim JY, Kleinman A, Basilico M. (eds.) Reimagining Global Health: An Introduction. University of California Press; 2013.

[15] John CC, Ayodo G, Musoke P. Successful global health research partnerships: What makes them work? Am J Trop Med Hyg. 2016; 94(1): 5–7. DOI: 10.4269/ajtmh.15-0611 26483123 PMC4710444

[16] Hedt-Gauthier B, Amoroso C, Warugaba C, et al. Building equity in the global health research agenda: The partners in health-harvard medical school research partnership in Rwanda. Ann Glob Heal. 2015; 81(1): 151–152. http://www.embase.com/search/results?subaction=viewrecord&from=export&id=L72073784. DOI: 10.1016/j.aogh.2015.02.848

[17] Morton B, Vercueil A, Masekela R, et al. Consensus statement on measures to promote equitable authorship in the publication of research from international partnerships. Anaesthesia. Published online 2021; 264–276. DOI: 10.1111/anae.15597 34647323 PMC9293237

[18] Abimbola S. The foreign gaze: Authorship in academic global health. BMJ Glob Heal. 2019; 4(5): 1–5. DOI: 10.1136/bmjgh-2019-002068 PMC6830280 31750005

[19] Adams LV, Wagner CM, Nutt CT, Binagwaho A. The future of global health education: training for equity in global health. BMC Med Educ. 2016; 16(1): 1–7. DOI: 10.1186/s12909-016-0820-0 27871276 PMC5117699

[20] Johnson SB. Advancing global health equity in the COVID-19 response: Beyond solidarity. J Bioeth Inq. Published online; 2020. DOI: 10.1007/s11673-020-10008-9 PMC7445720 32840837

[21] Balazs CL, Morello-Frosch R. The three R’s: How community based participatory research strengthens the rigor, relevance and reach of science. Env Justice. 2013; 6(1): 1–13. DOI: 10.1089/env.2012.0017 PMC3832061 24260590

[22] Sibanda A, Doctor HV. Measuring health gaps between the rich and the poor: A review of the literature and its implications for health research in Africa. J Public Health Africa. 2013; 4(1): 12–18. DOI: 10.4081/jphia.2013.e3 PMC5345422 28299092

[23] Zinsstag J, Schelling E, Waltner-Toews D, Tanner M. From “one medicine” to “one health” and systemic approaches to health and well-being. Prev Vet Med. 2011; 101(3–4): 148–156. DOI: 10.1016/j.prevetmed.2010.07.003 20832879 PMC3145159

